# Risk stratification for in-hospital mortality in sepsis-associated acute kidney injury patients receiving continuous renal replacement therapy: an interpretable, externally validated machine learning study

**DOI:** 10.1080/0886022X.2026.2677246

**Published:** 2026-06-17

**Authors:** Yao Zheng, Jian Gao, Tianfeng Hua, Wanguo Dong, Min Yang

**Affiliations:** ^a^ The Second Department of Critical Care Medicine, The Second Affiliated Hospital of Anhui Medical University, Hefei, P. R. China; ^b^ The Laboratory of Cardiopulmonary Resuscitation and Critical Care, The Second Affiliated Hospital of Anhui Medical University, Hefei, P. R. China

**Keywords:** Sepsis-associated acute kidney injury, continuous renal replacement therapy, in-hospital mortality, machine learning, prognostic model

## Abstract

Patients with sepsis-associated acute kidney injury (SA-AKI) requiring continuous renal replacement therapy (CRRT) have a high risk of in-hospital mortality, and early risk stratification may support timely clinical decision-making and efficient resource allocation. In this retrospective study, we developed and validated a prognostic model for SA-AKI patients receiving CRRT using data from the Medical Information Mart for Intensive Care IV (MIMIC-IV version 3.1 United States, 2008–2022) and the eICU Collaborative Research Database (eICU-CRD United States, 2014–2015), with external validation in an independent cohort from the intensive care unit of the Second Affiliated Hospital of Anhui Medical University (AYEFY-ICU China, 2021–2024). Candidate variables were selected using the least absolute shrinkage and selection operator (LASSO) and Boruta algorithms, and eight machine learning models were constructed and compared. Model interpretability was assessed using SHapley Additive exPlanations (SHAP). A total of 1,217 patients from the MIMIC-IV and eICU-CRD databases and 332 patients from the AYEFY-ICU cohort were included, and ten predictors were ultimately identified. Among the evaluated models, the gradient boosting machine (GBM) showed strong performance, with AUCs of 0.890, 0.756, and 0.752 in the training, internal validation, and external validation cohorts, respectively. In the external cohort, its performance was comparable to XGBoost and LightGBM without significant differences, with overlapping confidence intervals, while exceeding conventional scores (SOFA, SAPS II). SHAP analysis identified urine output, serum creatinine, and age as key predictors. This multicenter-derived GBM model may support early risk stratification and clinical decision-making in SA-AKI patients receiving CRRT.

## Graphical Abstract

**Figure:**
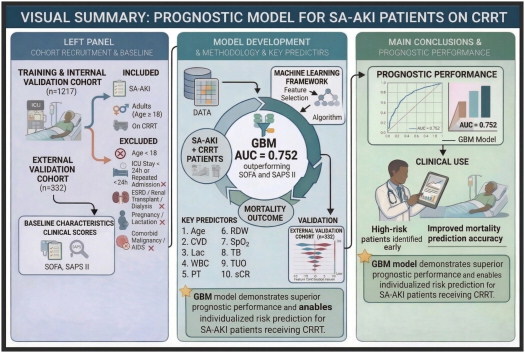

## 1. Introduction

Sepsis remains a major challenge in critical care medicine worldwide, accounting for approximately 20% of global deaths [1,2]. Acute kidney injury (AKI) is one of the most frequent and severe complications of sepsis, and over 70% of patients with sepsis develop AKI [3,4], Multiple pathophysiological mechanisms are involved in the development of sepsis-associated AKI (SA-AKI), including systemic and renal inflam­mation, altered microcirculatory and endothelial function, complement activation, renin–angiotensin–aldosterone system dysregulation, intra-renal shunting, mitochondrial dysfunction, and metabolic reprogramming [5,6]. These processes can result in renal tubular injury and functional impairment, leading to highly heterogeneous clinical presentations and outcomes in patients with SA-AKI.

For SA-AKI patients with hemodynamic instability, severe metabolic derangements, or fluid overload, continuous renal replacement therapy (CRRT) is an essential form of organ support [7]. SA-AKI patients receiving CRRT represent a distinct high-risk subgroup, with reported in-hospital mortality rates ranging from 30% to 53% [8,9]. Compared with other renal support therapies, CRRT has been shown to reduce inflammatory markers and improve organ dysfunction in patients with SA-AKI [10].

Nevertheless, these physiological benefits do not necessarily translate into improved survival. CRRT is associated with potential adverse effects, including catheter-related complications, hemodynamic instability, and variability in treatment delivery, which may offset its benefits [11]. In addition, extracorporeal clearance during CRRT can alter the pharmacokinetics of antibiotics and other critical medications. Consequently, previous studies have not consistently demonstrated a reduction in 90-day mortality with CRRT compared with standard strategies [12]. Moreover, the optimal timing of CRRT initiation in SA-AKI remains controversial, as early initiation may not improve clinical outcomes and could expose patients to unnecessary treatment in those with potential for renal recovery [13,14]. This inconsistency may reflect differences in patient selection, suggesting that only specific subgroups derive benefit from early CRRT initiation. Taken together, these findings suggest substantial heterogeneity in patient responses to CRRT. This heterogeneity is likely multifactorial and may be partly explained by differences in hemodynamic response trajectories during treatment, which have been shown to be closely associated with clinical outcomes [13]. Notably, a recent systematic review and meta-analysis suggested that the early initiation of CRRT may be associated with improved 28-day survival in patients with SA-AKI, particularly among those with Kidney Disease: Improving Global Outcomes (KDIGO) stage 2 disease and a Sequential Organ Failure Assessment (SOFA) score ≤12 [15].

Conventional severity scoring systems, such as SOFA and the Simplified Acute Physiology Score II (SAPS II), are widely used in the intensive care unit (ICU) for patients with sepsis. However, their predictive performance remains limited in this highly complex and heterogeneous population. Machine learning (ML) approaches can integrate high-dimensional clinical data and improve prognostic prediction by identifying complex, non-linear patterns [16]. Nevertheless, most existing ML studies have focused on the overall SA-AKI population, and only a few have specifically examined patients with AKI requiring CRRT [17–20]. Importantly, ML-based prognostic models specifically developed for the particularly high-risk subgroup of SA-AKI patients receiving CRRT remain scarce.

Therefore, this study aimed to develop and externally validate an interpretable ML-based model to predict in-hospital mortality among patients with SA-AKI receiving CRRT. The secondary objectives were to evaluate the model’s performance in terms of discrimination, calibration, and clinical utility, and to compare its prognostic value with conventional severity scoring systems. By integrating routinely collected clinical information using interpretable modeling approaches, we sought to facilitate early and individualized risk stratification, enhance prognostic assessment beyond conventional severity scores, and provide clinically meaningful insights into key factors associated with adverse outcomes in this vulnerable population. This could ultimately support more informed clinical assessment and management of critically ill patients with SA-AKI.

## 2. Methods

### 2.1. Data sources

This study was designed as a retrospective cohort study. The data used to create the training and internal validation cohorts were obtained from the Medical Information Mart for Intensive Care IV (MIMIC-IV, version 3.1) and the eICU Collaborative Research Database (eICU-CRD, version 2.0). The MIMIC-IV database contains detailed ICU admission data from Beth Israel Deaconess Medical Center between 2008 and 2022, while the eICU-CRD includes multicenter ICU data from 208 hospitals across the United States collected between 2014 and 2015 [21,22].

An independent external validation cohort was established using data from the intensive care unit of the Second Affiliated Hospital of Anhui Medical University (AYEFY-ICU). This cohort included patients with SA-AKI who received CRRT between January 2021 and December 2024.

### 2.2. Study population and definitions

Adult patients diagnosed with SA-AKI and receiving CRRT were eligible for inclusion. Detailed CRRT prescription data were available in the MIMIC-IV database. CRRT was typically initiated after ICU admission (median 48 h, IQR 21–96 h; see Results). In contrast, in the eICU-CRD, CRRT-related information was not consistently recorded at a granular level; therefore, detailed treatment parameters such as blood flow rate and effluent dose could not be reliably extracted.

Sepsis was defined according to the Third International Consensus Definitions for Sepsis and Septic Shock (Sepsis-3) as an increase in the SOFA score of at least two points in the presence of suspected or documented infection [1]. AKI was defined based on the KDIGO criteria as an increase in serum creatinine (sCR) of at least 0.3 mg/dL within 48 hours; an increase to at least 1.5 times the baseline sCR within the previous 7 days; or urine output of < 0.5 mL/kg/h for at least 6 hours [5].

Sepsis-associated acute kidney injury (SA-AKI) was defined as AKI occurring within 7 days after the diagnosis of sepsis, in accordance with the 28th Acute Disease Quality Initiative (ADQI) consensus [6].

Patients were excluded if they met any of the following criteria: age <18 years; repeated ICU admissions (only the first ICU admission was included); ICU length of stay <24 hours; missing outcome data; a history of renal transplantation or end-stage kidney disease; pregnancy or lactation; long-term maintenance dialysis; or a comorbid malignancy or acquired immunodeficiency syndrome (AIDS).

The primary outcome of this study was in-hospital mortality, defined as death before hospital discharge during the index admission. Secondary objectives were to evaluate the model’s performance against traditional severity scores and to assess feature contributions using SHAP analysis.

### 2.3. Data extraction and management

The following variables were extracted from each database: demographics, comorbidities, worst vital signs and laboratory values within the first 24 hours after ICU admission, severity scores (SOFA and SAPS II), and early therapeutic interventions. Detailed variable definitions and time windows are available in Supplementary Table S1.

Variables with more than 30% missing values were excluded. Outliers in continuous variables were considered implausible and treated as missing. Missing data were handled using multiple imputation by chained equations (MICE; 5 datasets, 10 iterations), with all candidate predictor variables included in the imputation model. Although the outcome variable had no missing values, it was also included as an auxiliary variable to preserve predictor–outcome associations. Estimates were pooled using Rubin’s rules. The proportion of missing data for each variable is presented in Supplementary Table S2.

### 2.4. Statistical analysis

Continuous variables were analyzed without categorization and summarized as medians with interquartile ranges, with no data-driven cutoffs applied. Group comparisons were performed using the Mann–Whitney U test for continuous variables and the chi-square test or Fisher’s exact test for categorical variables. All tests were two-sided, with *p* < 0.05 considered statistically significant. Analyses were performed using R software (version 4.2.3).

Patients from the MIMIC-IV and eICU-CRD databases were randomly divided into training and internal validation cohorts at a ratio of 7:3 for model development and validation. Candidate predictors were selected based on the intersection of variables identified by the least absolute shrinkage and selection operator (LASSO) regression method and the Boruta algorithm. Eight prediction models were subsequently developed, including logistic regression (LR), support vector machine (SVM), gradient boosting machine (GBM), neural network (NN), extreme gradient boosting (XGBoost), k-nearest neighbor (k-NN) model, adaptive boosting (AdaBoost), and Light Gradient Boosting Machine (LightGBM) model. Hyperparameters were optimized using grid search with 10-fold cross-validation for most models. For certain models, default parameter settings were retained when tuning did not result in meaningful performance improvement or to maintain model simplicity. (Supplementary Table S3).

Model discrimination was assessed using the area under the receiver operating characteristic curve (AUROC), along with the corresponding 95% confidence intervals. Overall predictive performance was evaluated using the Brier score. Calibration was assessed using calibration curves and the Hosmer–Lemeshow goodness-of-fit test. Decision curve analysis (DCA) was performed to evaluate the net clinical benefit of different models across a range of threshold probabilities. The optimal cutoff value was determined using the Youden index in the training cohort. This cutoff was then applied to the external cohorts, and sensitivity, specificity, positive predictive value (PPV), and negative predictive value (NPV) were calculated to assess classification performance.

External validation was conducted using the AYEFY-ICU cohort to assess model generalizability. SHAP analysis was used to interpret the model, quantifying how individual features positively or negatively contribute to the predicted risk of in-hospital mortality.

To minimize bias, standardized inclusion and data extraction procedures were applied across cohorts, with predictors restricted to the first 24-h worst values, and missing data and outliers handled using multiple imputation and predefined rules.

A web-based calculator was derived from the final model for individualized risk estimation. This study was reported in accordance with the STROBE and RECORD guidelines (Supplementary Table S4).

### 2.5. Ethics approval and consent to participate

Access to the MIMIC-IV and eICU-CRD databases was granted upon completion of the required training and certification (certification number: 63805862). All data extracted from these databases were de-identified, and informed consent was waived due to the retrospective nature of the study and the use of anonymized data. Collection and analysis of data for the external validation cohort were approved by the Ethics Committee of the Second Affiliated Hospital of Anhui Medical University (approval number: YX2025-055). The requirement for informed consent was waived by the Ethics Committee owing to the retrospective study design and minimal risk to participants. This study was conducted in accordance with the principles of the Declaration of Helsinki.

## 3. Result

### 3.1. Study population and baseline characteristics

**Figure 1.**
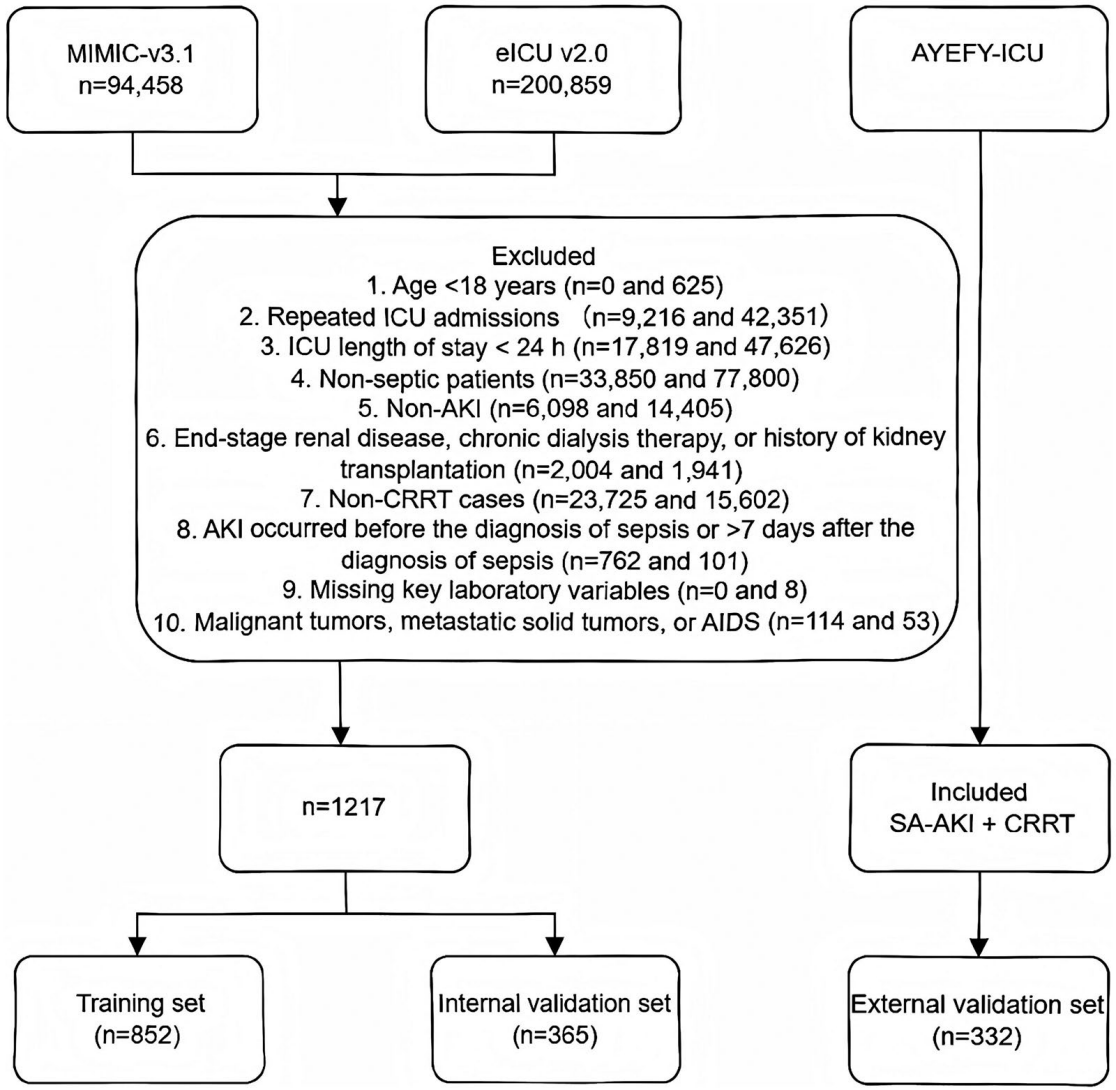
Flow diagram for patient selection and cohort conformation.

A total of 1,549 patients were included in the final analysis: 1,217 patients from the MIMIC-IV and eICU-CRD databases and 332 patients from the AYEFY-ICU external cohort. The recruitment and exclusion criteria leading to the final cohort are depicted in the flow diagram (Figure 1).

Baseline demographic and clinical characteristics of the cohort are detailed in Table 1. In-hospital mortality occurred in 627 patients (51.5%) in the overall cohort. In the MIMIC-IV cohort, the median time from ICU admission to CRRT initiation was 48 h (IQR 21–96 h). Overall, the median duration of CRRT was 69 h. The median blood flow rate was 150 mL/min, and the median prescribed CRRT dose was 24.1 mL/kg/h. In the eICU cohort, detailed CRRT prescription parameters were not consistently available. Non-survivors were older, with a higher prevalence of myocardial infarction and liver disease compared to survivors. They also required more vasopressors but fewer diuretics, and exhibited significantly higher SAPS II and SOFA scores.

**Table 1.** Baseline characteristics of survivors and non-survivors.

Characteristic	Total cohort (*n* = 1,217)	Survivors (*n* = 590)	Non-survivors (*n* = 627)	*P* value
**Demographics**				
Age, years	62 [52–73]	61 [51–70]	63[52–74]	0.004
Sex, male n (%)	706 (58.0)	348 (59.0)	358 (57.1)	0.543
Height, cm	170.0 [163.0–177.8]	170.0 [163.0–177.8]	170.0 [163.0–177.8]	0.634
Weight, kg	89.3 [73.6–107.3]	90.0 [73.15–110.0]	88.3 [73.9–106.45]	0.388
**Vital signs**				
Temperature, °C	36.8 [36.4–37.2]	36.8 [36.5–37.2]	36.8 [36.4–37.2]	0.072
Heart rate, beats/min	94.0 [81.0–106.0]	93.0 [80.0–105.0]	95.0 [82.0–107.0]	0.244
Respiratory rate, breaths/min	22.0 [19.0–26.0]	21.0 [18.0–24.0]	23.0 [19.0–26.0]	<0.001
Mean arterial pressure, mmHg	73.0 [68.0–78.0]	73.0 [69.0–78.0]	72.7 [68.0–77.8]	0.15
Peripheral oxygen saturation, %	96.0 [94.0–98.0]	96.0 [95.0–98.0]	96.0 [94.0–97.0]	<0.001
**Urine output**				
Total urine output, mL	2983.0 [623.0–10550.0]	5326.5 [1623.5–15838.3]	1376.0 [360.5–5664.5]	<0.001
**Blood gas and laboratory variables**				
Arterial pH	7.23 [7.13–7.31]	7.23 [7.15–7.31]	7.23 [7.12–7.31]	0.21
Lactate, mmol/L	3.8 [2.1–7.6]	3.3 [1.9–6.3]	4.3 [2.2–9.4]	<0.001
PaO₂, mmHg	76.0 [63.0–92.0]	76.0 [64.0–94.0]	75.0 [62.0–91.8]	0.193
PaCO₂, mmHg	45.2 [38.0–54.0]	45.0 [37.1–53.0]	46.0 [38.7–56.0]	0.055
Base excess, mmol/L	–9.0 [–14.0-–4.3]	−9.0 [−14.0-(−4.0)]	−9.0 [−14.0-(−4.9)]	0.569
Anion gap, mmol/L	19.0 [15.0–24.0]	19.0 [15.0–23.6]	20.0 [16.0–25.0]	0.019
WBC, ×10⁹/L	16.3 [11.2–23.2]	16.2 [11.2–22.7]	16.4 [11.2–23.6]	0.585
Hemoglobin, g/dL	9.2 [7.6–11.0]	9.3 [7.8–11.0]	9.0 [7.4–10.9]	0.004
Platelet count, ×10⁹/L	129.0 [70.0–199.0]	138.0 [81.0–202.8]	120.0 [62.5–197.0]	<0.001
RDW, %	15.9 [14.6–17.9]	15.7 [14.5–17.4]	16.3 [14.7–18.6]	<0.001
Creatinine, mg/dL	2.8 [1.8–4.2]	3.1 [1.9–4.7]	2.6 [1.7–3.8]	0.004
BUN, mg/dL	44.0 [28.0–71.0]	46.0 [31.0–72.8]	42.0 [26.0–68.0]	0.542
ALT, U/L	64.0 [30.0–260.0]	64.0 [30.0–235.5]	64.4 [29.5–286.5]	0.043
AST, U/L	141.0 [55.0–537.0]	135.5 [50.3–469.5]	150.0 [59.0–630.1]	0.102
ALP, U/L	102.0 [68.0–154.0]	98.0 [67.0–151.0]	104.0 [70.0–156.5]	<0.001
Total bilirubin, mg/dL	1.5 [0.7–3.9]	1.4 [0.7–3.1]	1.6 [0.8–4.9]	<0.001
PT, s	18.8 [14.8–25.6]	17.5 [14.4–22.6]	20.5 [15.3–29.9]	<0.001
APTT, s	43.7 [33.8–61.8]	40.8 [32.8–54.2]	47.7 [35.0–69.4]	<0.001
INR	1.7 [1.3–2.4]	1.6 [1.3–2.1]	1.9 [1.4–2.8]	0.445
Glucose, mg/dL	190.0 [145.0–267.0]	193.0 [148.3–263.3]	188.0 [142.0–271.0]	0.826
Potassium, mmol/L	4.9 [4.3–5.7]	4.9 [4.3–5.7]	4.8 [4.4–5.7]	0.208
Phosphate, mmol/L	5.6 [4.2–7.2]	5.5 [4.1–7.1]	5.7 [4.2–7.3]	0.018
Total calcium, mmol/L	7.5 [6.8–8.1]	7.4 [6.7–8.1]	7.6 [6.9–8.2]	0.572
Chloride, mmol/L	100.0 [95.0–104.0]	100.0 [95.0–104.0]	100.0 [95.0–105.0]	0.087
Sodium, mmol/L	135.0 [131.0–138.0]	135.0 [131.0–138.0]	135.0 [131.0–139.0]	0.044
Magnesium, mg/dL	1.81 [1.60–2.10]	1.80 [1.60–2.10]	1.90 [1.60–2.20]	<0.001
**Score**				
SAPS II score	55.0 [45.0–66.0]	52.5 [43.0–63.0]	57.0 [46.0–68.5]	<0.001
SOFA score	11.0 [8.0–13.0]	10.0 [7.0–13.0]	11.0 [8.0–14.0]	<0.001
**Comorbidities and therapies**				
Hypertension, n (%)	278 (22.8)	137 (23.2)	141 (22.5)	0.814
Diabetes mellitus, n (%)	439 (36.1)	227 (38.5)	212 (33.8)	0.102
Myocardial infarction, n (%)	220 (18.0)	85 (14.4)	135 (21.5)	0.002
Congestive heart failure, n (%)	401 (33.0)	187 (31.7)	214 (34.1)	0.4
Mild liver disease, n (%)	352 (28.9)	130 (22.0)	222 (35.4)	<0.001
Moderate-to-severe liver disease, n (%)	206 (16.9)	76 (12.9)	130 (20.7)	<0.001
Renal disease, n (%)	334 (27.4)	167 (28.3)	167 (26.6)	0.556
Chronic pulmonary disease, n (%)	272 (22.4)	132 (22.4)	140 (22.3)	1
Cerebrovascular disease, n (%)	132 (10.8)	54 (9.2)	78 (12.4)	0.08
Peripheral vascular disease, n (%)	164 (13.5)	72 (12.2)	92 (14.7)	0.239
Dementia, n (%)	16 (1.3)	9 (1.5)	7 (1.1)	0.708
Rheumatic disease, n (%)	40 (3.3)	15 (2.5)	25 (4.0)	0.211
Diuretic use, n (%)	52 (4.3)	33 (5.6)	19 (3.0)	0.039
Vasopressor use, n (%)	922 (75.8)	429 (72.7)	493 (78.6)	0.019
Antibiotic therapy, n (%)	987 (81.1)	470 (79.7)	517 (82.5)	0.241

Abbreviations: WBC, White Blood Cell; RDW, Red Blood Cell Distribution Width; BUN, Blood Urea Nitrogen; ALT, Alanine Aminotransferase; AST, Aspartate Aminotransferase; ALP, Alkaline Phosphatase; PT, Prothrombin Time; APTT, Activated Partial Thromboplastin Time; INR, International Normalized Ratio; SAPS II, Simplified Acute Physiology Score II; SOFA, Sequential Organ Failure Assessment.

### 3.2. Feature selection

**Figure 2.**
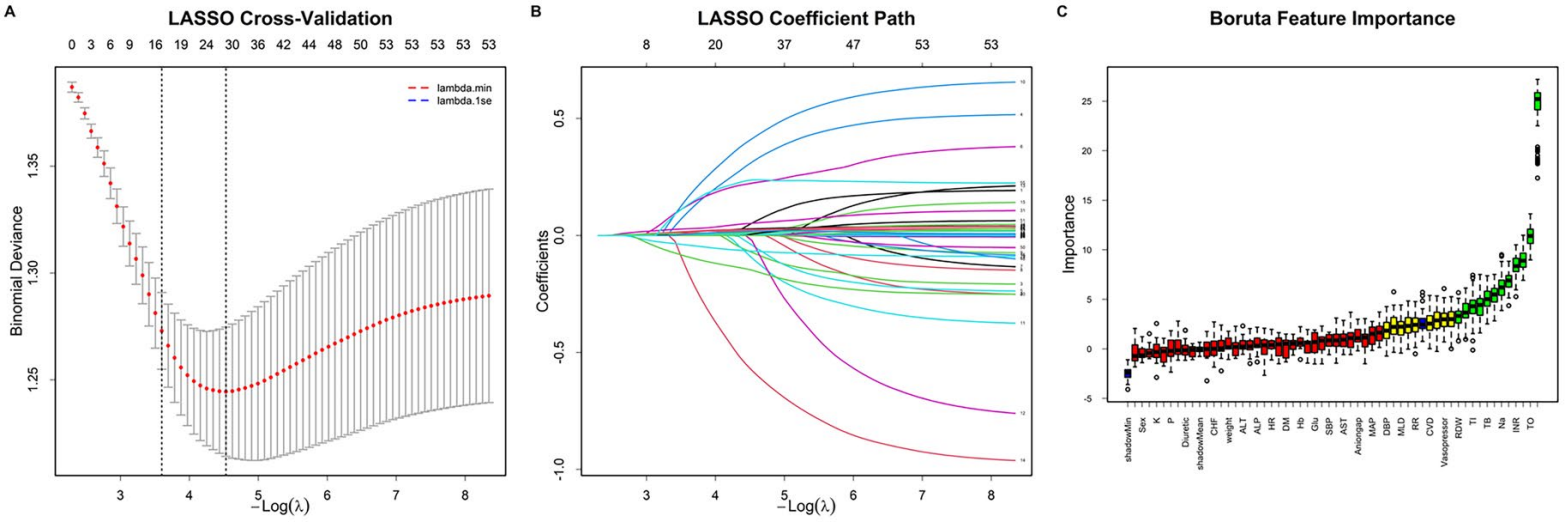
Feature selection using the LASSO regression and Boruta algorithms. (A) Cross-validation plot for the penalty term. The dashed lines represent the lambda.min and lambda.1se. (B) Plots for the LASSO regression coefficients over different values of the penalty parameter. (C) Feature importance ranking obtained using the Boruta algorithm. Variables were evaluated based on their relative importance compared with shadow features, and classified as confirmed (green), tentative (yellow), or rejected (red).

A combined feature selection strategy integrating LASSO regression and the Boruta algorithm was applied. A total of 55 candidate variables were initially included. LASSO regression and the Boruta algorithm each identified 16 variables, and the final feature set was defined as their intersection, yielding 10 predictors for model development (Figure 2). These predictors covered baseline comorbidities, markers of perfusion and metabolic disturbance, renal and hepatic dysfunction, and coagulation abnormalities. The detailed results of variables selected by each method and their overlap are provided in Supplementary Table S5.

### 3.3. Model development and internal validation

Eight ML models were developed and compared based on the ten selected predictors. The GBM model demonstrated more consistent discrimination across datasets, achieving an AUROC of 0.890 in the training cohort and 0.756 in the internal validation cohort (Table 2). In contrast, LightGBM and k-NN showed reduced performance in the internal validation cohort. Pairwise comparisons of AUROC using the DeLong test confirmed the superior performance of the GBM model.

**Table 2.** Performance of models in the training and internal validation sets, with pairwise AUROC comparison against the GBM model using DeLong test.

	Training set				Internal set				
Model	AUROC (95%CI)	Brier score	Sensitivity	Specificity	AUROC (95%CI)	Brier score	Sensitivity	Specificity	*p* (vs GBM)
LR	0.736 (0.703–0.770)	0.208	0.740	0.615	0.741 (0.691–0.791)	0.207	0.681	0.701	>0.05
SVM	0.723 (0.689–0.757)	0.224	0.538	0.782	0.726 (0.675–0.777)	0.226	0.574	0.751	>0.05
GBM	0.890 (0.869–0.911)	0.141	0.756	0.877	0.756 (0.707–0.804)	0.202	0.707	0.684	reference
XGBoost	0.808 (0.779–0.837)	0.249	0.722	0.758	0.688 (0.634–0.742)	0.250	0.601	0.667	0.003
k-NN	0.933 (0.918–0.948)	0.128	0.829	0.862	0.681 (0.627–0.735)	0.233	0.713	0.571	0.066
AdaBoost	0.675 (0.644–0.706)	0.239	0.494	0.801	0.665 (0.616–0.714)	0.246	0.415	0.847	0.002
LightGBM	0.927 (0.910–0.943)	0.111	0.845	0.857	0.660 (0.604–0.716)	0.265	0.612	0.644	0.006
NN	0.750 (0.718–0.783)	0.194	0.522	0.891	0.685 (0.630–0.739)	0.225	0.564	0.802	0.054
SOFA	0.576 (0.538–0.614)	–	–	–	0.535 (0.476–0.594)	–	–	–	<0.001
SAPS II	0.590 (0.552–0.628)	–	–	–	0.540 (0.481–0.600)	–	–	–	<0.001

Abbreviations: LR, logistic regression; SVM, support vector machine; GBM, gradient boosting machine; XGBoost, extreme gradient boosting; k-NN, k-nearest neighbor; AdaBoost, adaptive boosting; LightGBM, Light Gradient Boosting Machine; NN, neural network; SOFA, Sequential Organ Failure Assessment; SAPS II, Simplified Acute Physiology Score II.

Calibration analysis showed good agreement between the predicted and observed in-hospital mortality for the GBM model in the internal validation cohort. The calibration curves closely approximated the ideal 45-degree line, with a non-significant Hosmer–Lemeshow test (*p* = 0.129).

Conventional severity scores (SOFA and SAPS II) exhibited inferior discriminatory performance compared to the GBM model in the validation setting (Supplementary Figure S1).

Based on its stable discrimination and calibration performance, the GBM model was selected for further external validation. The optimal cutoff value (0.561) derived from the training cohort was subsequently applied in the external validation analysis.

### 3.4. External validation and clinical utility

**Figure 3.**
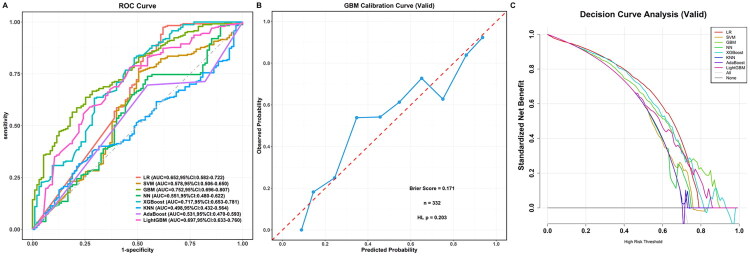
Discrimination, calibration, and clinical utility of prediction models in the external validation cohort. (A) Receiver operating characteristic (ROC) curves comparing the discriminative performance of the gradient boosting machine (GBM) model with other ML models and conventional severity scores. (B) Calibration curve of the GBM model, illustrating the agreement between predicted probabilities and observed in-hospital mortality; the dashed diagonal line represents ideal calibration. Hosmer–Lemeshow test p = 0.203. (C) Decision curve analysis (DCA) comparing the net clinical benefit of the GBM model, other ML models, and conventional severity scores across a range of threshold probabilities.

In the external validation cohort, with an in-hospital mortality rate of 65.4%, the GBM model demonstrated the highest discriminatory ability (AUROC = 0.752, 95% CI: 0.696–0.807). However, its performance was comparable to that of XGBoost and LightGBM, with no statistically significant differences according to the DeLong test (Supplementary Table S6). XGBoost and LightGBM showed moderate discrimination, whereas LR, SVM, k-NN, and AdaBoost had lower AUROC values (Figure 3A). The GBM model showed good calibration in the external validation cohort, with slight overestimation at higher predicted risk levels (Figure 3B). The Hosmer–Lemeshow test further supported good calibration (*p* = 0.203). In addition, the Brier score of the GBM model (0.17) was lower than that of the null model (0.25), indicating improved overall predictive performance.

DCA demonstrated that the GBM model provided a greater net benefit across a wide range of threshold probabilities than all other ML models and conventional severity scores (Figure 3C).

The predefined cutoff value (0.561) derived from the training cohort was applied to the external validation cohort. At this threshold, the GBM model achieved a sensitivity of 72%, specificity of 69%, PPV of 81.5%, and NPV of 54.7%, correctly classifying approximately 710 of 1,000 patients.

To facilitate clinical application, a simplified web-based calculator was developed to enable individualized risk estimation. The tool is publicly accessible at: https://gaojian.shinyapps.io/make_web/.

### 3.5. Model interpretability

**Figure 4.**
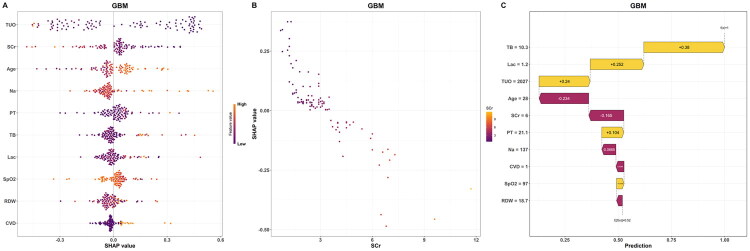
Model interpretability of the GBM model using SHAP analysis. (A) SHAP summary bar plot showing the global importance of the 10 selected predictors, ranked according to their mean absolute SHAP values, reflecting their overall contribution to in-hospital mortality prediction. (B) SHAP dependence plot illustrating the nonlinear relationship between sCR levels and predicted in-hospital mortality risk. Lower creatinine levels are associated with positive SHAP values, indicating an increased contribution to mortality risk, whereas this effect attenuates or reverses at higher creatinine levels. (C) SHAP waterfall plot for an individual patient, the yellow bar and purple bar represented a positive and negative contribution to in-hospital mortality, respectively. The cumulative contributions of individual predictors shift the model output from the baseline prediction to the final patient-specific predicted risk of in-hospital mortality.

SHAP analysis was performed to improve the interpretability of the final GBM model. Based on the mean absolute SHAP values, the variables that contributed most to predicting in-hospital mortality were, in descending order: total urine output (TUO), sCR, age, sodium (Na^+^), prothrombin time (PT), total bilirubin (TB), lactate (Lac), oxygen saturation (SpO_2_), red blood cell distribution width (RDW), and cerebrovascular disease (CVD) (see Supplementary Figure S2). Among these predictors, CVD, advanced age, elevated lactate, increased RDW, higher TB, prolonged PT, and hypernatremia were associated with an increased risk of mortality, whereas higher TUO, greater SpO_2_, and higher sCR were associated with a reduced predicted risk (Figure 4A).

SHAP dependence plots further demonstrated a non-linear relationship between sCR levels and predicted in-hospital mortality. Lower creatinine levels were associated with positive SHAP values, indicating an increased contribution to mortality risk. However, this effect was attenuated or even reversed at higher creatinine levels (see Figure 4B). SHAP dependence plots for additional predictors are provided in Supplementary Figure S3. At the individual patient level, SHAP waterfall plots illustrated the cumulative contributions of multiple predictors to the final risk estimate. These plots clearly distinguish factors that increase or decrease the predicted risk, thereby providing an intuitive visualization of how the model output evolved from the baseline prediction to the patient-specific mortality risk (see Figure 4C).

## 4. Discussion

In this study, we observed an in-hospital mortality exceeding 50% among SA-AKI patients treated with CRRT, consistent with previous reports [9]. This highlights that SA-AKI patients requiring CRRT represent a high-risk and heterogeneous subgroup, in whom outcomes vary widely despite similar indications for organ support. Against this background, we developed and externally validated an interpretable prognostic model using routinely collected clinical parameters obtained during the first 24 h of ICU admission. The model demonstrated good discrimination and calibration across the internal cohorts and maintained acceptable and clinically meaningful performance in the external cohort, enabling early and clinically meaningful risk stratification. A predefined cutoff further facilitated practical classification of high-risk patients. Importantly, rather than replacing existing severity scoring systems, this model provides complementary prognostic information to enhance risk assessment. As this cutoff was derived from the training cohort, recalibration or threshold adjustment may be needed in other populations, and site-specific validation is recommended before clinical implementation.

Several prognostic models have been proposed for mortality prediction in critically ill or AKI populations. Most previous studies have focused on general ICU or AKI populations without paying specific attention to patients receiving CRRT, and have reported model performance mainly within derivation cohorts or using internal validation only [17–19]. In contrast, our study focused specifically on SA-AKI patients undergoing CRRT-a relatively understudied, high-risk, and heterogeneous population-and demonstrated consistent predictive performance across development, internal validation, and external cohorts. Although a modest decline in discrimination was observed in the external cohort, the GBM model maintained good calibration and showed better discrimination than conventional severity scores such as SOFA and SAPS II. Beyond differences in patient case-mix, this attenuation in performance may also reflect heterogeneity in healthcare systems and CRRT treatment practices across institutions and geographic regions. Variations in CRRT initiation timing, anticoagulation strategies, fluid management, and ICU care pathways may influence both patient trajectories and clinical outcomes, thereby affecting model transportability across cohorts. The clinical utility of the model was further supported by decision curve analysis, and a predefined risk threshold of 0.561 was used to define high-risk patients, enabling clinically interpretable risk stratification. To facilitate bedside application, these components were integrated into a simplified web-based calculator for individualized risk estimation. Although this tool does not fully reproduce the complexity of the original GBM model, it provides a pragmatic approximation for clinical use when full model deployment is not feasible, thereby supporting the translation of model-derived insights into routine practice.

From a pathophysiological perspective, the selected predictors primarily reflected three major domains: perfusion and microcirculation, inflammation–coagulation imbalance, and physiological reserve.

Perfusion and microcirculation status were found to be the most influential dimension. Total urine output was identified as the single most important predictor, integrating renal perfusion, glomerular filtration, and systemic hemodynamic status, and serving as a direct indicator of effective organ perfusion and residual kidney function. Lower urine output was strongly associated with an increased risk of mortality [23,24]. Elevated serum sodium and lactate levels were also associated with an increased mortality risk, reflecting disturbances in fluid balance and impaired tissue perfusion. In particular, lactate is a well-established marker of microcirculatory dysfunction and disordered oxygen utilization in septic shock, whereas hypernatremia often indicates free water deficit and persistent circulatory inadequacy [25–28].

The inflammation–coagulation domain was primarily characterized by PT, TB, and RDW. Together, these variables reflect endothelial dysfunction, hepatic impairment, and sustained inflammatory activation, which are key components of sepsis-associated coagulopathy [29–33]. Physiological reserve was captured by age, SpO_2_, and a history of cerebrovascular disease. Older age and preexisting cerebrovascular comorbidity indicate reduced compensatory capacity, whereas higher SpO_2_ reflects preserved cardiopulmonary reserve [34,35]. These findings are consistent with previous evidence indicating that baseline illness severity frequently overrides treatment-related factors in determining outcomes among SA-AKI patients receiving CRRT [36].

A paradoxical association was observed between serum creatinine and mortality: higher creatinine levels were linked to a lower predicted risk. However, this should not be interpreted as a protective effect. Lower creatinine levels in critically ill patients may indicate hemodilution, reduced muscle mass, impaired creatinine generation, or diminished physiological reserve, all of which are associated with poor prognosis [37,38]. Early CRRT initiation in fluid-overloaded or hemodynamically unstable patients may further weaken the relationship between creatinine and outcomes, contributing to the observed “creatinine paradox”. In addition, because serum creatinine measurements were obtained during the early ICU period surrounding CRRT initiation, the observed association may partially reflect timing-related and treatment-related confounding rather than a direct protective biological effect.

Notably, most of the predictors in our study overlap with the components of conventional severity scoring systems. This indicates that these scores capture core dimensions of organ dysfunction in critically ill patients. Rather than replacing existing scoring systems, our model builds upon them by preserving continuous clinical information and incorporating complementary markers that are underweighted in traditional scores. In particular, lactate and RDW provide additional pathophysiological insight that is not explicitly represented in conventional scores, reflecting microcirculatory dysfunction and systemic inflammation, respectively [39,40]. These mechanisms are central to mortality risk in SA-AKI patients receiving CRRT, yet they are underweighted in conventional severity scoring systems.

The relative contribution of individual predictors in the GBM model should not be interpreted as equivalent to point assignments in conventional scoring systems. Unlike traditional scores, which categorize continuous variables and assume uniform risk within thresholds, the ML model retains continuous information and recognizes context-dependent, non-linear effects [41]. SHAP analysis further enhances interpretability by quantifying feature contributions at the individual level, without implying causal relationships.

The improved prognostic performance of the GBM model likely reflects a physiologically coherent representation of disease severity, rather than simply an increased number of predictors. Randomized trials evaluating CRRT timing and intensity have not consistently demonstrated survival benefits in SA-AKI populations [42], possibly due to underlying heterogeneity in baseline risk. Our findings suggest that patients with similar indications for CRRT may have markedly different predicted mortality risks, highlighting the potential value of risk-stratified study designs to improve the interpretability of treatment effects.

**Figure 5.**
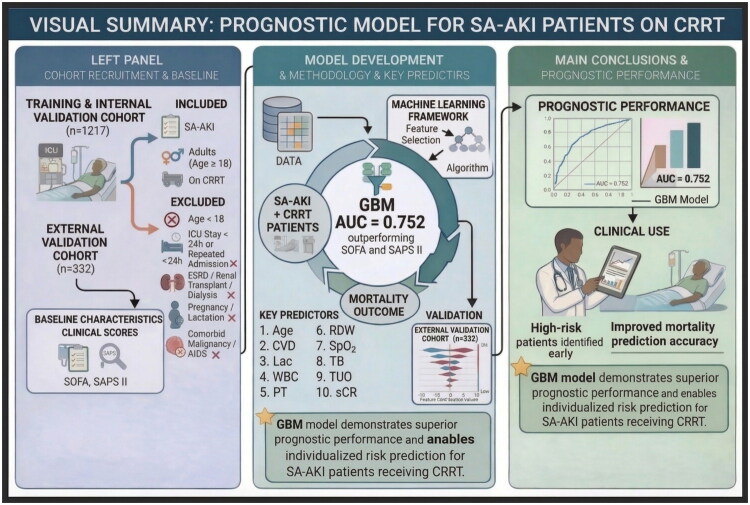
Prognostic model for SA-AKI patients receiving CRRT. A total of 1,549 patients were included, comprising a development cohort (n = 1,217) and an external validation cohort (n = 332). A gradient boosting machine (GBM) model was developed using clinical variables reflecting perfusion, inflammation, and organ function. In the external validation cohort, the model demonstrated good discrimination (AUC = 0.752) and outperformed conventional severity scores. The model enables risk stratification and identification of high-risk patients with SA-AKI receiving CRRT.

Clinically, this model is not intended to guide individual decisions regarding CRRT initiation. Instead, it may support risk stratification, facilitate risk-adjusted outcome comparisons, and inform the design of future observational studies and clinical trials. Future work should focus on prospective validation across diverse healthcare settings and integration with dynamic patient data. The overall study workflow and clinical utility of our model are synthesized in Figure 5.

Several limitations to this study that should be acknowledged. Firstly, the retrospective design restricts the ability to draw causal inferences, and residual confounding cannot be completely ruled out. Secondly, while the model was trained and internally validated using two large public ICU databases and further tested in an independent real-world cohort, all the data were derived from critically ill patients in tertiary care settings, which may limit its generalizability. In addition, the external validation cohort was derived from a single center and differed systematically from the development cohorts in patient characteristics and clinical practice patterns. Variations in CRRT initiation timing and fluid management strategies, together with inter-institutional differences in data collection, may have contributed to the observed attenuation in model performance. Moreover, detailed CRRT prescription variables, including modality, dose, and precise timing, were not fully captured in the present study. Such granular treatment information was available in the MIMIC-IV database but not consistently recorded in the eICU database, limiting a more comprehensive characterization of CRRT exposure across cohorts. Thirdly, although predictor variables were restricted to the first 24 h after ICU admission to reduce potential information leakage, some patients may have initiated CRRT within this time window. Therefore, a small degree of temporal overlap cannot be completely excluded. However, given that CRRT was typically initiated after 24 h in the MIMIC-IV cohort, the impact of this issue is likely to be limited. Finally, SHAP-based interpretations enhance model interpretability but do not imply causality and should be considered hypothesis-generating. Prospective multicenter studies with standardized data collection are warranted to further validate and refine the model.

In conclusion, we developed and externally validated an interpretable prognostic model for in-hospital mortality in SA-AKI patients receiving CRRT, who are a high-risk and heterogeneous population. The model demonstrated improved discrimination and calibration compared with conventional severity scores, with acceptable discrimination and calibration maintained during external validation across independent cohorts. By integrating routinely available clinical variables, the model provides clinically interpretable risk stratification that may complement existing severity scoring systems. Although not intended to guide individual treatment decisions, it offers a framework for future prospective studies and risk-stratified evaluation of CRRT outcomes.

## Supplementary Material

Revised Supplementary Material.docx

## Data Availability

The data that support the findings of this study are available from publicly accessible databases, including the MIMIC-IV database (https://doi.org/10.13026/7vcr-e114) and the eICU Collaborative Research Database (https://doi.org/10.13026/C2WM1R), both hosted on PhysioNet. Access requires completion of the relevant training and data use agreements. Additional data were obtained from a local institutional database. These data are not publicly available due to privacy and ethical restrictions but are available from the corresponding author upon reasonable request and with institutional approval.
