# Supplementary material for: Risk stratification for in-hospital mortality in sepsis-associated acute kidney injury patients receiving continuous renal replacement therapy: an interpretable, externally validated machine learning study
Source: Ren Fail. 2026 Jun 17;48(1):2677246. doi: 10.1080/0886022X.2026.2677246 (PMC13276809; doi:10.1080/0886022X.2026.2677246)
Supplement: Revised Supplementary Material.docx [file IRNF_A_2677246_SM0499.docx]

Supplementary Materials

Supplementary F
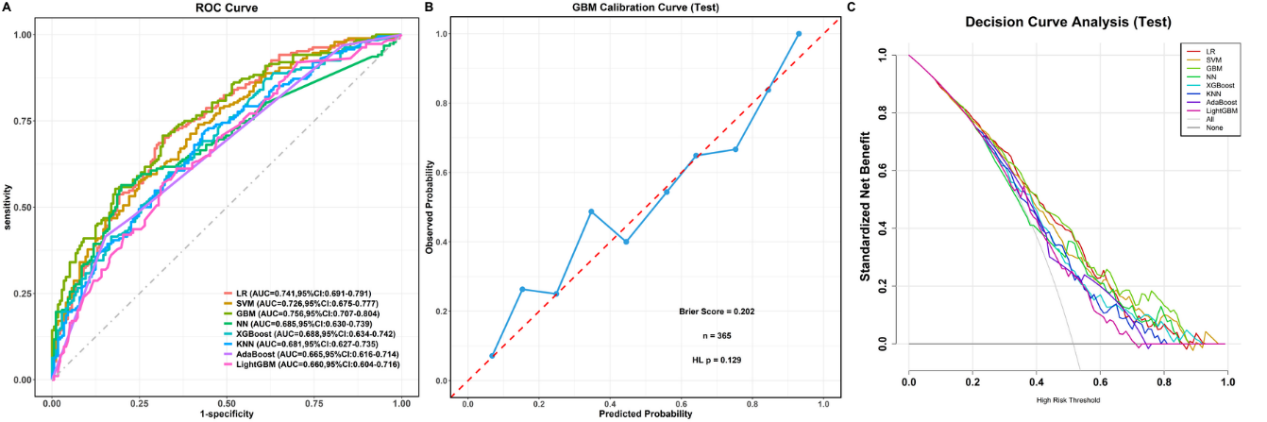
igures:

Supplementary Figure S1. Discrimination, calibration, and clinical utility of prediction models in the internal validation cohort.

(A) Receiver operating characteristic (ROC) curves comparing the discriminative performance of the gradient boosting machine (GBM) model with other ML models and conventional severity scores.

(B) Calibration curve of the GBM model, illustrating the agreement between predicted probabilities and observed in-hospital mortality; the dashed diagonal line represents ideal calibration. Hosmer–Lemeshow test p = 0.129.

(C) Decision curve analysis (DCA) comparing the net clinical benefit of the GBM model, other ML models, and conventional severity scores across a range of threshold probabilities.


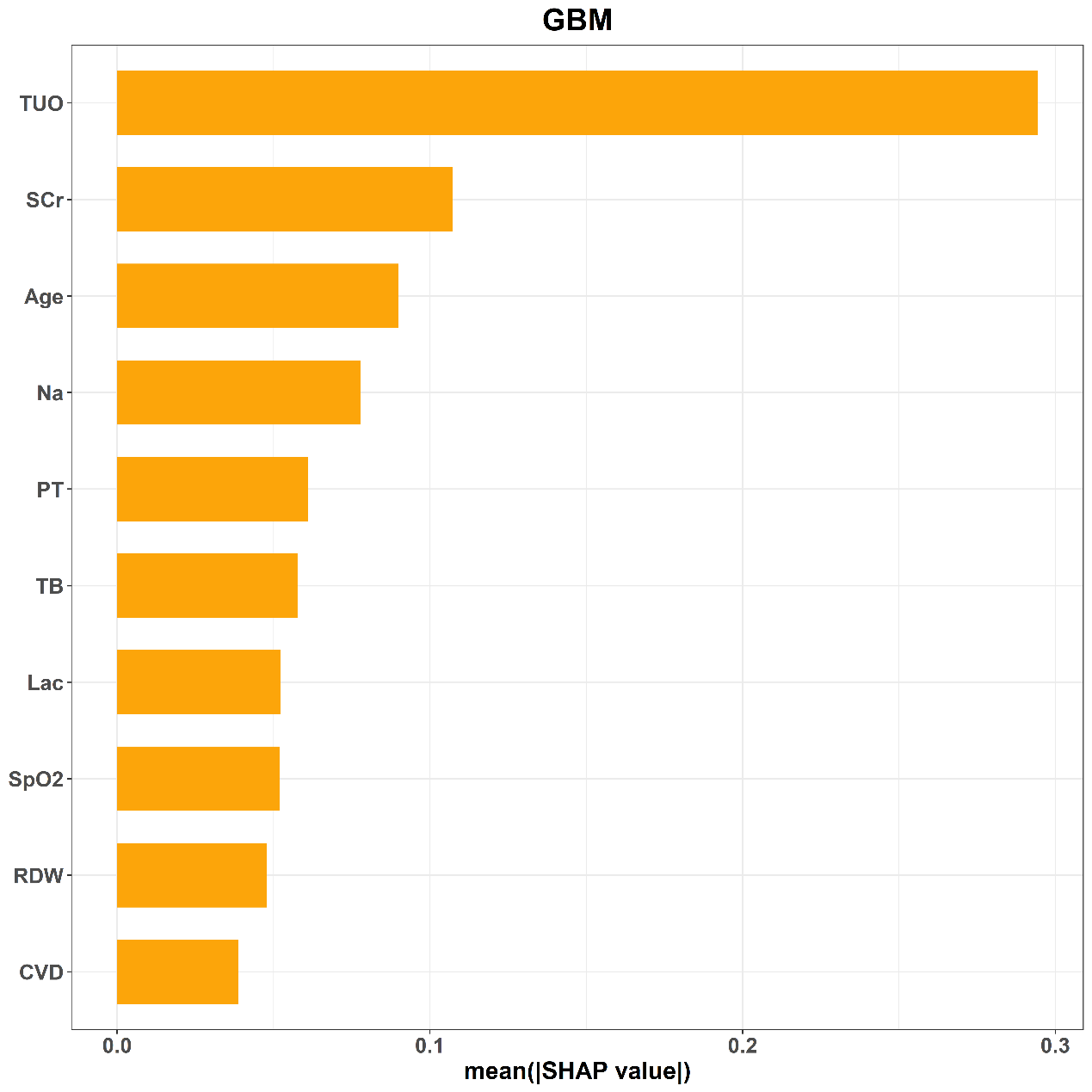


Supplementary Figure S2. Feature importance ranking plot of the GBM model.

TUO: total urine output; SCr: serum creatinine; Na: sodium; PT: prothrombin time; TB: total bilirubin; Lac: lactate; SpO₂: oxygen saturation; RDW: red blood cell distribution width; CVD: cerebrovascular disease.


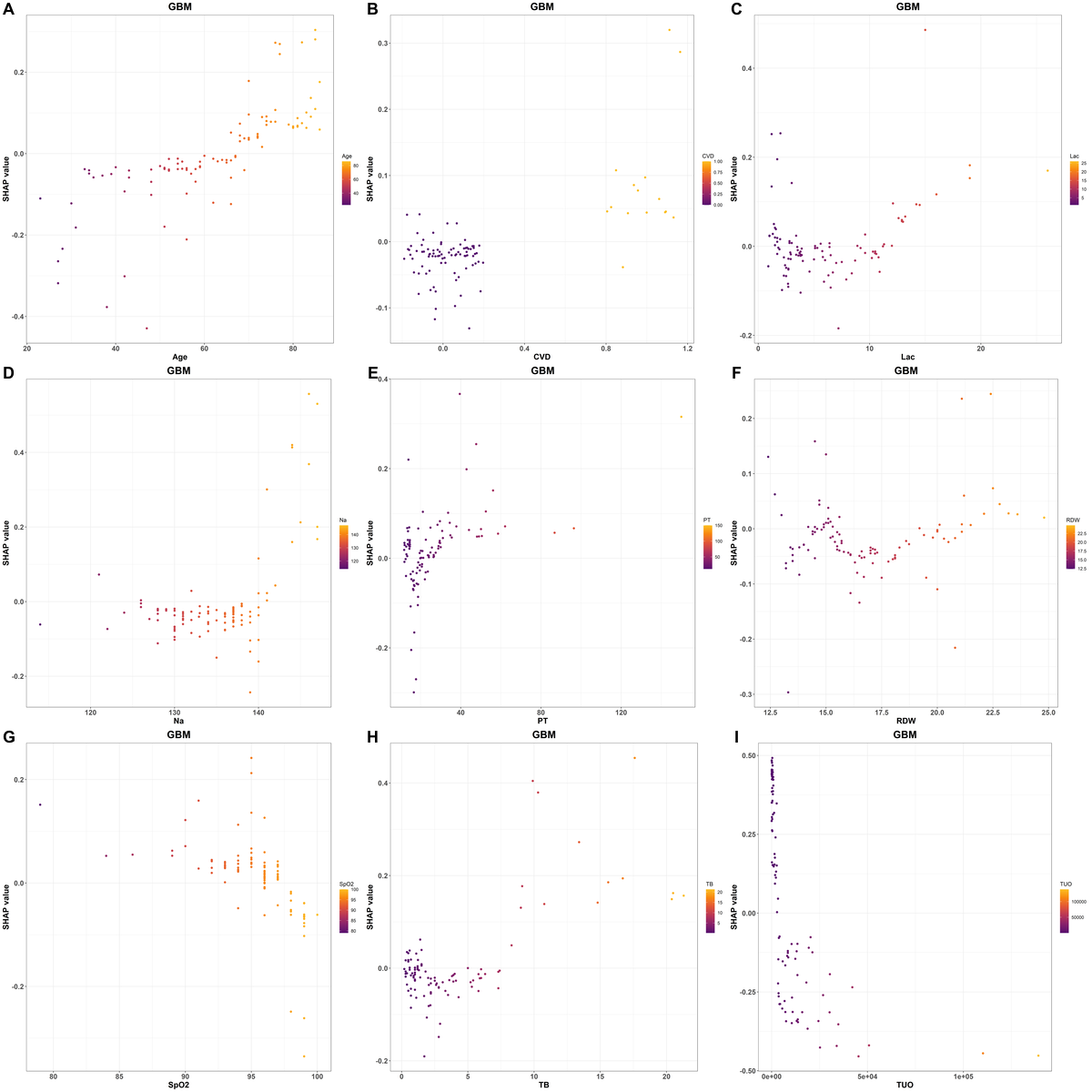


Supplementary Figure S3. SHAP dependence plot of the GBM model.

1. Age; (B) CVD; (C) Lac; (D) Na; (E) PT; (F) RDW; (G) SpO_2_; (H) TB; (I) TUO.

Supplementary Tables:

Supplementary Table S1. Definitions of all variables and time windows for model development.

Continuous physiological and laboratory variables were extracted from the electronic health record and summarized as the worst value within the first 24 hours after ICU admission. Comorbidities were defined based on documented diagnoses in the medical record. Severity scores were calculated according to standard definitions.

| Variable | Definition | Time window |
| --- | --- | --- |
| In-hospital mortality | hospital discharge status = death | index admission |
| Sepsis | sepsis-3 | index admission |
| AKI | KDIGO criteria | index admission |
| SA-AKI | 28^th^ ADQI | within 7 days post-sepsis diagnosis |
| CRRT | procedure/chart events | ICU stay |
| Sex | recorded in EHR | at ICU admission |
| Age | recorded in EHR | at ICU admission |
| Height | recorded in EHR | at ICU admission |
| Weight | recorded in EHR | at ICU admission |
| HTN | hypertension | before or at index hospital admission |
| DM | diabetes mellitus | before or at index hospital admission |
| AMI | acute myocardial infarction | before or at index hospital admission |
| CHF | congestive heart failure | before or at index hospital admission |
| MLD | mild liver disease | before or at index hospital admission |
| SLD | severe liver disease | before or at index hospital admission |
| CKD | chronic kidney disease | before or at index hospital admission |
| COPD | chronic obstructive pulmonary disease | before or at index hospital admission |
| CVD | cerebrovascular disease | before or at index hospital admission |
| PVD | peripheral vascular disease | before or at index hospital admission |
| DEM | dementia | before or at index hospital admission |
| RD | rheumatic disease | before or at index hospital admission |
| Diuretic | any use within first 24h | within first 24h after ICU admission |
| Vasopressor | any use within first 24h | within first 24h after ICU admission |
| Antibiotic | any use within first 24h | within first 24h after ICU admission |
| SAPSII | worst physiology | within first 24h after ICU admission |
| SOFA | worst values | within first 24h after ICU admission |
| HR | max | within first 24h after ICU admission |
| RR | max | within first 24h after ICU admission |
| SBP | min | within first 24h after ICU admission |
| DBP | min | within first 24h after ICU admission |
| MAP | min | within first 24h after ICU admission |
| SpO2 | min | within first 24h after ICU admission |
| T | max | within first 24h after ICU admission |
| PH | min | within first 24h after ICU admission |
| Lac | max | within first 24h after ICU admission |
| PaO2 | min | within first 24h after ICU admission |
| PaCO2 | max | within first 24h after ICU admission |
| BE | min | within first 24h after ICU admission |
| WBC | max | within first 24h after ICU admission |
| Hb | min | within first 24h after ICU admission |
| PLT | min | within first 24h after ICU admission |
| RDW | max | within first 24h after ICU admission |
| SCr | max | within first 24h after ICU admission |
| BUN | max | within first 24h after ICU admission |
| ALT | max | within first 24h after ICU admission |
| AST | max | within first 24h after ICU admission |
| ALP | max | within first 24h after ICU admission |
| TB | max | within first 24h after ICU admission |
| Glucose | min | within first 24h after ICU admission |
| Potassium | max | within first 24h after ICU admission |
| Sodium | max | within first 24h after ICU admission |
| Chloride | max | within first 24h after ICU admission |
| Calcium | min | within first 24h after ICU admission |
| Magnesium | min | within first 24h after ICU admission |
| Phosphorus | min | within first 24h after ICU admission |
| Aniongap | max | within first 24h after ICU admission |
| TI | max | within first 24h after ICU admission |
| TO | min | within first 24h after ICU admission |
| TUO | min | within first 24h after ICU admission |

Abbreviations: HTN: Hypertension; DM: Diabetes Mellitus; AMI: Acute Myocardial Infarction; CHF: Congestive Heart Failure; MLD: Mild Liver Disease; SLD: Severe Liver Disease; CKD: Chronic Kidney Disease; COPD: Chronic Obstructive Pulmonary Disease; CVD: Cerebrovascular Disease; PVD: Peripheral Vascular Disease; DEM: Dementia; RD: Rheumatic Disease; SAPSII: Simplified Acute Physiology Score II; SOFA: Sequential Organ Failure Assessment; height: Height; weight: Weight; TI: Total Input; TO: Total Output; TUO: Total Urine Output; T: Temperature; HR: Heart Rate; RR: Respiratory Rate; SBP: Systolic Blood Pressure; DBP: Diastolic Blood Pressure; MAP: Mean Arterial Pressure; SpO2: Peripheral Oxygen Saturation; PH: Potential of Hydrogen; Lac: Lactic Acid; PaO2: Partial Pressure of Oxygen; PaCO2: Partial Pressure of Carbon Dioxide; BE: Base Excess; Aniongap: Anion Gap; WBC: White Blood Cell; Hb: Hemoglobin; PLT: Platelet; RDW: Red Blood Cell Distribution Width; SCr: Serum Creatinine; BUN: Blood Urea Nitrogen; ALT: Alanine Aminotransferase; AST: Aspartate Aminotransferase; ALP: Alkaline Phosphatase; TB: Total Bilirubin; PT: Prothrombin Time; APTT: Activated Partial Thromboplastin Time; INR: International Normalized Ratio.

Supplementary Table S2：Missing data summary.

| Variable | Missing rate (%) |
| --- | --- |
| Result | 0 |
| Sex | 0 |
| HTN | 0 |
| DM | 0 |
| AMI | 0 |
| CHF | 0 |
| MLD | 0 |
| SLD | 0 |
| CKD | 0 |
| COPD | 0 |
| CVD | 0 |
| PVD | 0 |
| DEM | 0 |
| RD | 0 |
| Diuretic | 0 |
| Vasopressor | 0 |
| Antibiotic | 0 |
| Age | 0 |
| SAPSII | 0 |
| SOFA | 0 |
| height | 5.916 |
| weight | 0 |
| TI | 15.859 |
| TO | 2.794 |
| TUO | 4.273 |
| T | 25.719 |
| HR | 0.247 |
| RR | 2.629 |
| SBP | 9.449 |
| DBP | 9.449 |
| MAP | 9.121 |
| SpO2 | 0.329 |
| PH | 12.325 |
| Lac | 17.502 |
| PaO2 | 12.325 |
| PaCO2 | 12.818 |
| BE | 19.145 |
| Aniongap | 5.834 |
| WBC | 0.822 |
| Hb | 0.822 |
| PLT | 0.986 |
| RDW | 2.794 |
| SCr | 0.493 |
| BUN | 0.411 |
| ALT | 12.901 |
| AST | 12.901 |
| ALP | 12.818 |
| TB | 13.476 |
| PT | 9.039 |
| APTT | 13.476 |
| INR | 6.984 |
| Glucose | 0.493 |
| Potassium | 0.329 |
| Phosphorus | 8.217 |
| Calcium | 2.465 |
| Chloride | 0.329 |
| Sodium | 0.329 |
| Magnesium | 5.423 |

Supplementary Table S3. Hyperparameter settings of the eight machine learning models used in this study.

| Model | Hyperparameters |
| --- | --- |
| Logistic Regression | Default parameters using the glm function; maximum likelihood estimation; no additional hyperparameters were tuned |
| SVM | kernel = radial basis function (RBF); sigma = 0.001; cost (C) = 0.09 |
| Gradient Boosting | n.trees = 100; interaction.depth = 3; shrinkage = 0.1; n.minobsinnode = 5 |
| Neural Network (NN) | size = 6; decay = 0.6 |
| XGBoost | nrounds = 1000; max_depth = 3; eta = 0.1; gamma = 0.5; colsample_bytree = 0.8; min_child_weight = 1; subsample = 0.6 |
| KNN | kmax = 12; distance = 1; kernel = "optimal" |
| AdaBoost | mfinal = 100; maxdepth = 1; coeflearn = "Zhu" |
| LightGBM | objective = binary; metric = auc; min_data_in_leaf = 20; learning_rate = 0.05; num_threads = 2; force_col_wise = TRUE; nrounds = 1000; early_stopping_rounds = 10 |

Supplementary Table S4. The RECORD statement – checklist of items, extended from the STROBE statement, that should be reported in observational studies using routinely collected health data.

|  | Item No. | STROBE items | Location in manuscript where items are reported | RECORD items | Location in manuscript where items are reported |
| --- | --- | --- | --- | --- | --- |
| Title and abstract | | | | | |
|  | 1 | (a) Indicate the study’s design with a commonly used term in the title or the abstract (b) Provide in the abstract an informative and balanced summary of what was done and what was found | (a) Abstract (Page 1);  (b) Abstract (Background, Methods, Results, Conclusions). | RECORD 1.1: The type of data used should be specified in the title or abstract. When possible, the name of the databases used should be included.  RECORD 1.2: If applicable, the geographic region and timeframe within which the study took place should be reported in the title or abstract.  RECORD 1.3: If linkage between databases was conducted for the study, this should be clearly stated in the title or abstract. | RECORD 1.1: Abstract (Data sources: MIMIC-IV, eICU-CRD, and AYEFY-ICU cohort).  RECORD 1.2: Abstract, Methods section (data source description including geographic region and timeframe)  RECORD 1.3: Not applicable (no database linkage was performed) |
| Introduction | | | | | |
| Background rationale | 2 | Explain the scientific background and rationale for the investigation being reported | Introduction: paragraphs 1–4. |  |  |
| Objectives | 3 | State specific objectives, including any prespecified hypotheses | Introduction: paragraph 5. |  |  |
| Methods | | | | | |
| Study Design | 4 | Present key elements of study design early in the paper | Abstract and Methods (2.1 Data Sources). |  |  |
| Setting | 5 | Describe the setting, locations, and relevant dates, including periods of recruitment, exposure, follow-up, and data collection | Methods: 2.2 Study population and Definitions. |  |  |
| Participants | 6 | *(a) Cohort study* - Give the eligibility criteria, and the sources and methods of selection of participants. Describe methods of follow-up  *Case-control study* - Give the eligibility criteria, and the sources and methods of case ascertainment and control selection. Give the rationale for the choice of cases and controls  *Cross-sectional study* - Give the eligibility criteria, and the sources and methods of selection of participants  *(b) Cohort study* - For matched studies, give matching criteria and number of exposed and unexposed  *Case-control study* - For matched studies, give matching criteria and the number of controls per case | (a)Methods: 2.1 Data Sources, 2.2 Study population and Definitions, 2.3 Data Extraction and Management.  (b)Not applicable (no matching was performed in this cohort study). | RECORD 6.1: The methods of study population selection (such as codes or algorithms used to identify subjects) should be listed in detail. If this is not possible, an explanation should be provided.  RECORD 6.2: Any validation studies of the codes or algorithms used to select the population should be referenced. If validation was conducted for this study and not published elsewhere, detailed methods and results should be provided.  RECORD 6.3: If the study involved linkage of databases, consider use of a flow diagram or other graphical display to demonstrate the data linkage process, including the number of individuals with linked data at each stage. | RECORD 6.1: Methods (2.2 Study population and Definitions).  RECORD 6.2: Methods (2.2 Study population and Definitions, previously validated definitions with supporting references).  RECORD 6.3: Not applicable (no database linkage was performed); study flow diagram provided in Figure 1. |
| Variables | 7 | Clearly define all outcomes, exposures, predictors, potential confounders, and effect modifiers. Give diagnostic criteria, if applicable. | Methods: 2.2 Study population and Definitions; 2.3 Data Extraction and Management. | RECORD 7.1: A complete list of codes and algorithms used to classify exposures, outcomes, confounders, and effect modifiers should be provided. If these cannot be reported, an explanation should be provided. | RECORD 7.1: Methods (2.2 Study population and Definitions; 2.3 Data Extraction and Management; 2.4 Statistical analysis) and Supplementary Table S5. |
| Data sources/ measurement | 8 | For each variable of interest, give sources of data and details of methods of assessment (measurement).  Describe comparability of assessment methods if there is more than one group | Methods: 2.3 Data Extraction and Management. |  |  |
| Bias | 9 | Describe any efforts to address potential sources of bias | Methods: 2.4 Statistical analysis. |  |  |
| Study size | 10 | Explain how the study size was arrived at | Methods: 2.2 Study population and Definitions; 2.3 Data Extraction and Management, 2.4 Statistical analysis. |  |  |
| Quantitative variables | 11 | Explain how quantitative variables were handled in the analyses. If applicable, describe which groupings were chosen, and why | Methods: 2.4 Statistical analysis. |  |  |
| Statistical methods | 12 | (a) Describe all statistical methods, including those used to control for confounding  (b) Describe any methods used to examine subgroups and interactions  (c) Explain how missing data were addressed  (d) *Cohort study* - If applicable, explain how loss to follow-up was addressed  *Case-control study* - If applicable, explain how matching of cases and controls was addressed  *Cross-sectional study* - If applicable, describe analytical methods taking account of sampling strategy  (e) Describe any sensitivity analyses | (a) Methods: 2.4 Statistical analysis.  (b) Not applicable (no subgroup or interaction analyses were performed).  (c) Methods: 2.3 Data Extraction and Management.  (d) *Cohort study* Not applicable (no longitudinal follow-up loss in retrospective database cohort).  (e) Methods: 2.4 Statistical analysis. |  |  |
| Data access and cleaning methods |  | .. |  | RECORD 12.1: Authors should describe the extent to which the investigators had access to the database population used to create the study population.  RECORD 12.2: Authors should provide information on the data cleaning methods used in the study. | RECORD 12.1: Methods: 2.5 Ethics approval and consent to participate.  RECORD 12.2: Methods: 2.3 Data Extraction and Management. |
| Linkage |  | .. |  | RECORD 12.3: State whether the study included person-level, institutional-level, or other data linkage across two or more databases. The methods of linkage and methods of linkage quality evaluation should be provided. | RECORD 12.3: Not applicable (no person-level, institutional-level, or cross-database linkage was performed) |
| Results | | | | | |
| Participants | 13 | (a) Report the numbers of individuals at each stage of the study (*e.g.*, numbers potentially eligible, examined for eligibility, confirmed eligible, included in the study, completing follow-up, and analysed)  (b) Give reasons for non-participation at each stage.  (c) Consider use of a flow diagram | (a) Results: Figure 1 Study flow diagram;  (b) 2.2 Study population and Definitions;  (c) Figure 1 (Study flow diagram). | RECORD 13.1: Describe in detail the selection of the persons included in the study (*i.e.,* study population selection) including filtering based on data quality, data availability and linkage. The selection of included persons can be described in the text and/or by means of the study flow diagram. | RECORD 13.1: 2.2 Study population and Definitions; Results: Figure 1 Study flow diagram. |
| Descriptive data | 14 | (a) Give characteristics of study participants (*e.g.*, demographic, clinical, social) and information on exposures and potential confounders  (b) Indicate the number of participants with missing data for each variable of interest  (c) *Cohort study* - summarise follow-up time (*e.g.*, average and total amount) | (a) Results: Table 1;  (b) Supplementary Table S1;  (c) Not applicable – outcome was in-hospital mortality assessed during index hospitalization (fixed follow-up window). |  |  |
| Outcome data | 15 | *Cohort study* - Report numbers of outcome events or summary measures over time  *Case-control study* - Report numbers in each exposure category, or summary measures of exposure  *Cross-sectional study* - Report numbers of outcome events or summary measures | Results: 3.1 Study population and baseline characteristics; 3.4 External validation and clinical utility. |  |  |
| Main results | 16 | (a) Give unadjusted estimates and, if applicable, confounder-adjusted estimates and their precision (e.g., 95% confidence interval). Make clear which confounders were adjusted for and why they were included  (b) Report category boundaries when continuous variables were categorized  (c) If relevant, consider translating estimates of relative risk into absolute risk for a meaningful time period | (a) Not applicable (This study focused on predictive modeling rather than causal inference; therefore, no confounder-adjusted effect estimates were derived.)  (b) Not applicable (No continuous variables were categorized in the primary analysis.)  (c) Results: 3.4 External validation and clinical utility. |  |  |
| Other analyses | 17 | Report other analyses done—e.g., analyses of subgroups and interactions, and sensitivity analyses | Not applicable. |  |  |
| Discussion | | | | | |
| Key results | 18 | Summarise key results with reference to study objectives | Discussion: Final paragraph. |  |  |
| Limitations | 19 | Discuss limitations of the study, taking into account sources of potential bias or imprecision. Discuss both direction and magnitude of any potential bias | Discussion: Limitation paragraph. | RECORD 19.1: Discuss the implications of using data that were not created or collected to answer the specific research question(s). Include discussion of misclassification bias, unmeasured confounding, missing data, and changing eligibility over time, as they pertain to the study being reported. | Discussion: Limitation paragraph. |
| Interpretation | 20 | Give a cautious overall interpretation of results considering objectives, limitations, multiplicity of analyses, results from similar studies, and other relevant evidence | Main Discussion section (integrated interpretation across paragraphs, before Conclusion) |  |  |
| Generalisability | 21 | Discuss the generalisability (external validity) of the study results | Discussion section (interpretation, limitations, and generalisability). |  |  |
| Other Information | | | | | |
| Funding | 22 | Give the source of funding and the role of the funders for the present study and, if applicable, for the original study on which the present article is based | End of the manuscript (Funding statement section). |  |  |
| Accessibility of protocol, raw data, and programming code |  | .. |  | RECORD 22.1: Authors should provide information on how to access any supplemental information such as the study protocol, raw data, or programming code. | End of manuscript (Data availability statement). |

Supplementary Table S5: A total of 55 candidate variables were evaluated. LASSO and Boruta each selected 16 variables, with 10 overlapping variables included in the final model.

| Variable | Boruta | LASSO | Final model |
| --- | --- | --- | --- |
| Age | ✔ | ✔ | ✔ |
| CVD | ✔ | ✔ | ✔ |
| TUO | ✔ | ✔ | ✔ |
| SpO2 | ✔ | ✔ | ✔ |
| Lac | ✔ | ✔ | ✔ |
| RDW | ✔ | ✔ | ✔ |
| sCR | ✔ | ✔ | ✔ |
| TB | ✔ | ✔ | ✔ |
| PT | ✔ | ✔ | ✔ |
| Sodium | ✔ | ✔ | ✔ |
| Vasopressor | ✔ | ✘ | ✘ |
| TI | ✔ | ✘ | ✘ |
| TO | ✔ | ✘ | ✘ |
| PH | ✔ | ✘ | ✘ |
| BE | ✔ | ✘ | ✘ |
| INR | ✔ | ✘ | ✘ |
| AMI | ✘ | ✔ | ✘ |
| MLD | ✘ | ✔ | ✘ |
| Diuretic | ✘ | ✔ | ✘ |
| Respiratory rate | ✘ | ✔ | ✘ |
| Calcium | ✘ | ✔ | ✘ |
| Magnesium | ✘ | ✔ | ✘ |

Supplementary Table S6. Performance of models in the external validation cohort with AUROC comparisons against the GBM model.

| Model | AUROC | Brier | Sensitivity | Specificity | *p* (vs GBM) |
| --- | --- | --- | --- | --- | --- |
| GBM | 0.752(0.696-0.807) | 0.171 | 0.637 | 0.747 | reference |
| LR | 0.652(0.582-0.722) | 0.237 | 0.680 | 0.605 | 0.115 |
| SVM | 0.578(0.506-0.650) | 0.261 | 0.759 | 0.495 | <0.001 |
| XGBoost | 0.717(0.653-0.781) | 0.249 | 0.827 | 0.516 | >0.05 |
| k-NN | 0.498(0.432-0.564) | 0.350 | 0.683 | 0.221 | <0.001 |
| AdaBoost | 0.531(0.470-0.593) | 0.366 | 0.696 | 0.453 | <0.001 |
| LightGBM | 0.697(0.633-0.760) | 0.239 | 0.781 | 0.537 | >0.05 |
| NN | 0.551(0.480-0.622) | 0.276 | 0.720 | 0.480 | <0.001 |
| SOFA | 0.657(0.590-0.725) | - | - | - | <0.001 |
| SAPS II | 0.589(0.521-0.656) | - | - | - | <0.001 |
